# 3D marker-controlled watershed for kidney segmentation in clinical CT exams

**DOI:** 10.1186/s12938-018-0456-x

**Published:** 2018-02-27

**Authors:** Wojciech Wieclawek

**Affiliations:** Department of Informatics and Medical Equipment, Faculty of Biomedical Engineering, Silesian University of Technology, Roosevelta 40, 41-800 Zabrze, Poland

**Keywords:** Computed tomography, Segmentation, Watershed transform, Mathematical morphology, Markers, Abdomen, Kidney

## Abstract

**Background:**

Image segmentation is an essential and non trivial task in computer vision and medical image analysis. Computed tomography (CT) is one of the most accessible medical examination techniques to visualize the interior of a patient’s body. Among different computer-aided diagnostic systems, the applications dedicated to kidney segmentation represent a relatively small group. In addition, literature solutions are verified on relatively small databases. The goal of this research is to develop a novel algorithm for fully automated kidney segmentation. This approach is designed for large database analysis including both physiological and pathological cases.

**Methods:**

This study presents a 3D marker-controlled watershed transform developed and employed for fully automated CT kidney segmentation. The original and the most complex step in the current proposition is an automatic generation of 3D marker images. The final kidney segmentation step is an analysis of the labelled image obtained from marker-controlled watershed transform. It consists of morphological operations and shape analysis. The implementation is conducted in a MATLAB environment, Version 2017a, using i.a. Image Processing Toolbox. 170 clinical CT abdominal studies have been subjected to the analysis. The dataset includes normal as well as various pathological cases (agenesis, renal cysts, tumors, renal cell carcinoma, kidney cirrhosis, partial or radical nephrectomy, hematoma and nephrolithiasis). Manual and semi-automated delineations have been used as a gold standard. Wieclawek Among 67 delineated medical cases, 62 cases are ‘Very good’, whereas only 5 are ‘Good’ according to Cohen’s Kappa interpretation. The segmentation results show that mean values of Sensitivity, Specificity, Dice, Jaccard, Cohen’s Kappa and Accuracy are 90.29, 99.96, 91.68, 85.04, 91.62 and 99.89% respectively. All 170 medical cases (with and without outlines) have been classified by three independent medical experts as ‘Very good’ in 143–148 cases, as ‘Good’ in 15–21 cases and as ‘Moderate’ in 6–8 cases.

**Conclusions:**

An automatic kidney segmentation approach for CT studies to compete with commonly known solutions was developed. The algorithm gives promising results, that were confirmed during validation procedure done on a relatively large database, including 170 CTs with both physiological and pathological cases.

## Background

Design of systems dedicated to computer-aided diagnostic (CAD) and medical image analysis has been a meaningful research area exploited for many years. This applies to various imaging modalities (X-ray, CT, MRI, OCT, ultrasound, etc.) as well as different parts of the human body. There is no effective and universal approach to segmentation of every medical image or even every anatomical structure. Therefore, research focused on such solutions is still justified.

Urology is one of the many research areas. Among various urological preventive or diagnostic test are CT examinations (apart from standard techniques: kidney X-rays or USG). CT scans of the kidneys can provide more detailed information related to injuries, kidney diseases, etc. They are expected to detect tumors or other lesions, obstructive conditions such as kidney stones, congenital anomalies, polycystic kidney disease, accumulation of fluid around the kidneys, and the location of abscesses. Thus, computer systems aiding urological diagnosis and treatment require kidney segmentation as a first step of many high-level processing tasks. This step often precedes volume measurement or abnormalities detection (i.e. cyst, tumor, etc.). Several approaches for kidney segmentation in CT studies are presented in the following section.

### State-of-the-art

Simple segmentation steps including region growing technique, gradient and edge-based segmentation, or others basic transformations (i.e. mathematical morphology operations) are implemented in kidney segmentation methods [1, 2]. More robust approaches are also employed. A level set deformable model has been extended to a stochastic speed function guided level set model [3, 4] and tested on 21 cases. The manual selection of seed points makes this technique insufficient for clinical implementation. A more complex methodology based on 3D shape-constrained graph cut method has been developed by Chen et al. [5] and evaluated on kidney donors. Similarly, comprehensive analysis consisting of two stages is presented in [6]. The rough segmentation is based on a kernel fuzzy C-means algorithm with spatial information and then a refined segmentation is implemented with an improved GrowCut algorithm.

An automated segmentation of poor and noisy images with low spatial resolution in the coronal and axial planes is based on a statistical approach [7]. Therefore, the authors adopt a deformable model, which uses not only the gray value of the target, but also statistical information of the shapes [8]. Their model is defined by the NURBS surface [9] in order to achieve easy manipulation and representation of a smooth shapes.

The kidney segmentation in MRI images is also addressed in the literature [10–12]. The two-phase genetic algorithm [10] as well as the detection of Maximally Stable Temporal Volume [11] have been developed. The MSTV approach exploits both 3D spatial correlation among voxels and temporal dynamics for each voxel to provide a reliable segmentation resistant to noise from surrounding tissues and kidney shape variations. This solution is a result of dynamic contrast-enhanced MRI images [12].

The data base, employed for evaluation, is limited to normal cases [2–4, 6] only or extended to selected pathologies, including tumor, cyst, ureter obstruction, atrophic change of renal parenchyma, or mild hydronephrosis [1]. A careful selection of cases results in a relatively high evaluation rate (accuracy) that ranges between 70.5 and 99.76%.

Kidney segmentation can also be applied as an intermediate step in a more complex procedure, such as cyst detection [13] or renal cortex segmentation [5]. Both cases refer to a small group of applications related to the specific pathology.

Recently, multi-organ segmentation techniques of the abdomen structures have been reported. Kidney extraction is one of the processing steps. The method presented in [14] is based on a hierarchical atlas registration and weighting scheme that generates target specific priors from an atlas database. The final segmentation is obtained by applying an automatically learned intensity model in a graph-cuts optimization step, incorporating high-level spatial knowledge. The proposition of a general framework of multi-organ segmentation which effectively incorporates interrelations among multiple organs and easily adapts to various imaging conditions without the need for supervised intensity information has been discussed in [15]. It consist of modeling the conditional shape and location priors and organ correlation graph analysis.

As shown above, the available solutions mainly focus on physiological cases or are dedicated to one specific pathology. It has been found that there is no versatile approach, which would work effectively in various situations and conditions. The methodology presented in this paper is meant to bridge this gap.

The aim of this study is to develop a new, fully automated kidney segmentation method able to extract normal as well as abnormal kidneys with no restriction on pathologies. The methodology employs a new marker generation approach for watershed transform. The method provides correct results for a variety of renal pathologies. Both kidneys are always segmented excluding agenesis, nephrectomy or resection cases [1–6]. A set of 170 CT studies have been subjected to the evaluation analysis. The majority of cases (158) are pathological, including agenesis, atrophy, nephrolithiasis, renal cysts, tumors, renal cell carcinoma, kidney cirrhosis, focal lesions, nephrostomy and partial or radical nephrectomy or resection. To the author’s best knowledge, segmentation of both kidneys in normal and pathological cases has not be reported so far [7–9].

## Methods

### Database

The testing database contained 170 volumetric abdomen CTs (including 89 female and 81 male). Medical examinations were carried out in years 2008–2013 by the Department and Institute of Medical Radiology and Radiodiagnosis in Zabrze, Medical University of Silesia, Poland. The Clinical Research Ethics Committee waived the need for the approval because anonymous clinical data was released from the hospital database. The medical protocol specified neither the size of axial section nor the range of abdomen (i.e. body range). Therefore, CT series consist of 33–337 slices (126 slices on average) of the resolution of 512 $\times$ 512 pixels. Other parameters of the CTs are: minimum voxel size 0.41 $\times$ 0.41 $\times$ 0.63 mm, maximum voxel size 0.98 $\times$ 0.98 $\times$ 5 mm, mean voxel size 0.75 $\times$ 0.75 $\times$ 2.5 mm and 32-bits depth. The scanning protocol always included pre-contrast phase, arterial phase, portal venous phase, and sometimes delayed phase. In the current study portal venous phase was used.

**Fig. 1:**
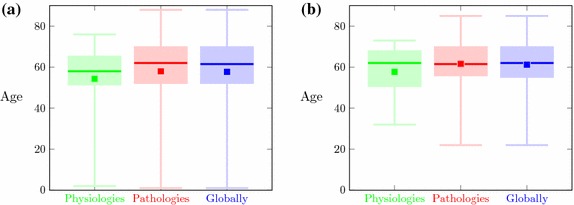
Patient age: (green) physiological cases, (red) pathological cases, (blue) globally **a** for all 170 cases, **b** for 60 delineated cases. The boxes are spanned between the first and third quartiles of age value, the lower and the upper whiskers are a minimum and a maximum age value, respectively and isolated points are a mean of age value

The image data is summarized in Table 1. The first row presents all medical cases available in database, including 170 CT examinations. Whereas, the second row contains cases (67 CTs) with ground truth delineations (manual or semi-automatic). Since in several cases more than one pathology occurs, the overall number of pathologies exceeds the number of exams. The next two rows shows the distribution of pathologies separately for manual or semi-automatic delineation. Notice that the sum of these numbers does not equal the number pf all delineated cases. Patient age statistics are shown in Fig. 1. Physiological cases are marked using green, pathological cases using red, while whole cases using blue colors.

**Table 1:**
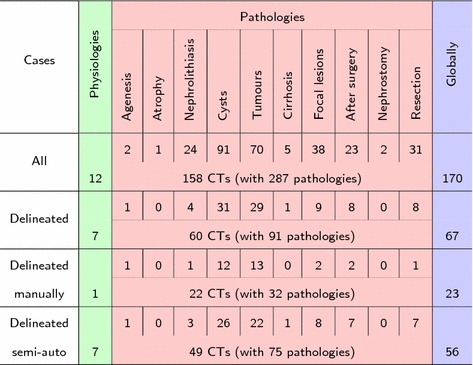
Medical cases in database

### Image analysis

**Fig. 2:**
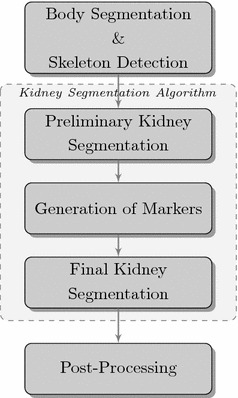
Workflow

The proposed fully-automatic kidney segmentation algorithm consists of several steps (Fig. 2). The workflow starts with body segmentation and skeleton detection procedure, which detects the abdominal contour. As a result, a region of interest is obtained. Then, a two-stage kidney segmentation followed by a post-processing procedure are applied. The following subsections present these steps in details.

### Body segmentation and skeleton detection

The reduction of the overall CT study starts with a removal of the area outside the patient’s body. At this stage, a small object removal is followed by binarization, which extracts voxels with a positive HU (hounsfield units [16]) value. Then, a rough location of a landmark region is needed in order to indicate the kidney position.

Initially, the lungs are used as landmark [13]. Despite the high accuracy of their segmentation, the final results strongly depend on patient’s position during the medical examination. A non-standard positioning may yield a mislocation of the kidney and result in a missegmentation.

In the current study, the skeleton detection precedes the main segmentation procedure and serves as a reference area. At this stage binarization is employed again. Yet, the threshold value corresponds to the HU of the bone structures [16]. The improvement of thresholding has been obtained by some morphological operations (i.e. opening), as well as 2D analysis of the transverse and sagittal planes.

**Fig. 3:**
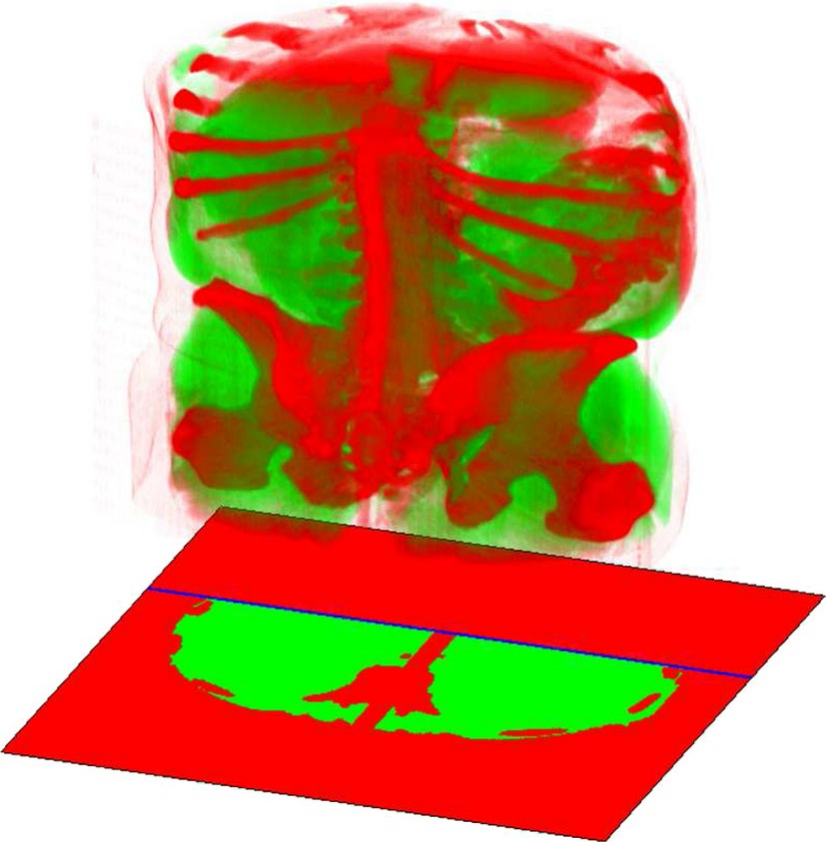
Patient body and skeleton segmentation

Due to the kidneys’ anatomical location, 30% of the abdomen can be removed [7]. This straightens a part of the patient-border line (blue line in Fig. 3). Removed area is marked in red, while the region subjected to further processing is highlighted in green.

### Kidney segmentation

In further processing, two anatomical features are employed. The first one is related to the kidney brightness, which is of approximately 30 HU for most abdominal CTs [16]. However, since the analyzed CT series are contrast-enhanced, a higher value range (0–200 HU) has been assumed. In further processing the image is referred to as *I*(*x*, *y*, *z*).

**Fig. 4:**
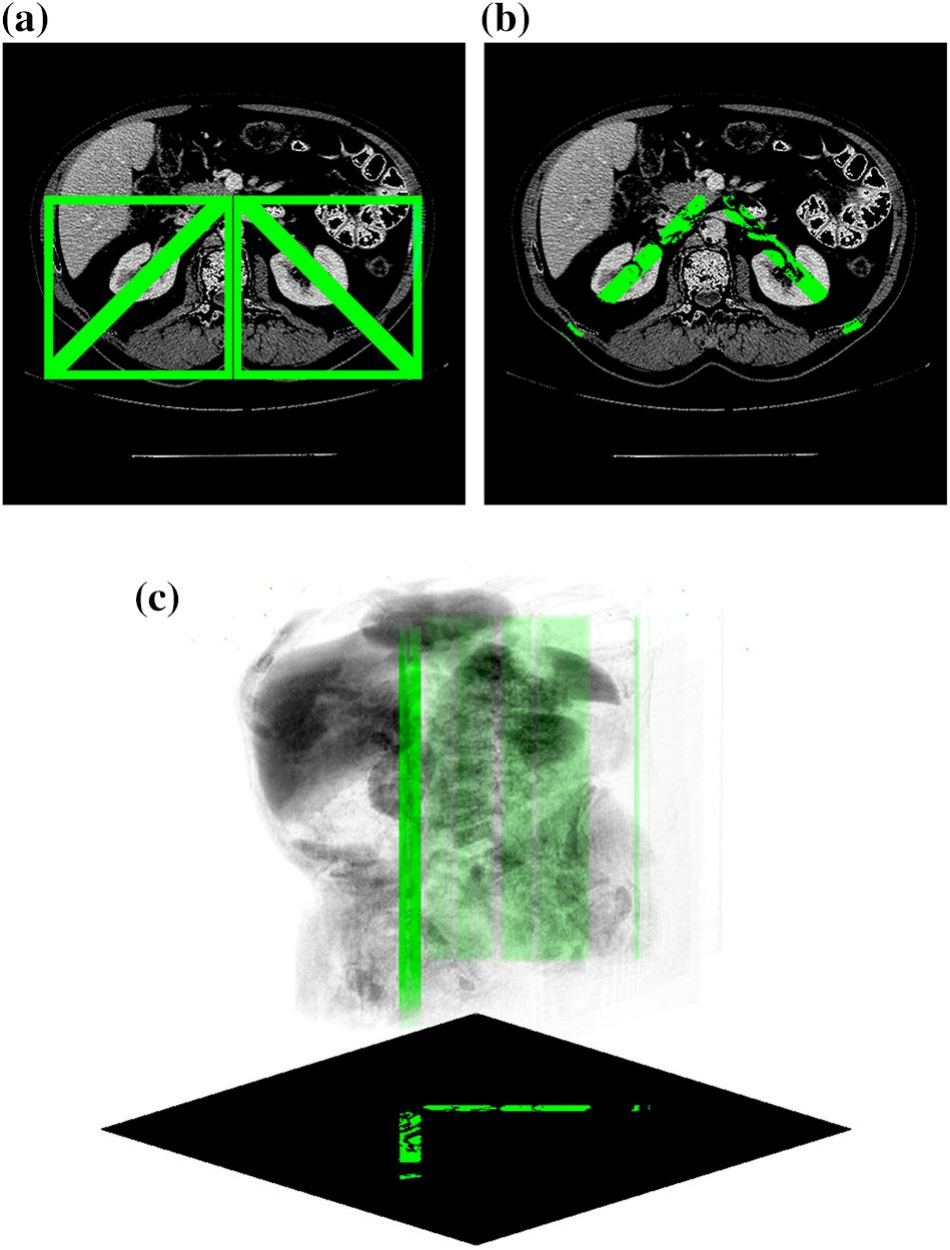
Binary masks facilitate kidney segmentation: **a** mask location, **b** voxels indicated by mask, **c** 3D mask view

The second feature deals with an anatomical constraint associated with geometric kidney location. Based on the body and skeleton view, the smallest bounding box containing the area for further analysis is defined (Fig. 4). Then, the central axis of the bounding box is found and brightened along the spine position. It divides the bounding box into two smaller boxes, containing the left and right kidney, respectively (Fig. 4a). Their diagonals intersect each kidney indicating their initial location (Fig. 4a). Moreover, in further analysis only nonzero voxels from *I*(*x*, *y*, *z*) indicated by the mask are considered (Fig. 4b).

This initial mask permits two areas to be defined. One reflects the kidney, whereas the other indicates the background. In further processing (i.e. “Preliminary kidney segmentation”) these areas are referred to as object marker and background marker, respectively.

#### Preliminary kidney segmentation

The binary mask presented in Fig. 4c indicates a large number of voxels constituting the kidneys. The sum of mean value and standard deviation of nonzero voxels along the diagonal is used as a *h* value in the *HMAX* transform:

1 $$\begin{aligned} HMAX_h \left( I\left( x,y,z\right) \right) = R_I^\delta \left( I\left( x,y,z\right) -h\right) , \end{aligned}$$

that smooths the brightness of kidney voxels in *I*(*x*, *y*, *z*), by suppressing all maxima of the intensity value below the *h* level. The $R_I^\delta \left( \bullet \right)$ is a morphological reconstruction by dilation, i.e.:

2 $$\begin{aligned} R_m^\delta \left( I\left( x,y,z\right) \right) = \delta _m^i \left( I\left( x,y,z\right) \right) , \end{aligned}$$

defined as an iterative geodesic dilation:

3 $$\begin{aligned} \delta _m^1 \left( I\left( x,y,z\right) \right) = \delta _{B_1} \left( I\left( x,y,z\right) \right) \cap m\left( x,y,z\right) = \min \left\{ \delta _{B_1} \left( I\left( x,y,z\right) \right) , m\left( x,y,z\right) \right\} , \end{aligned}$$

and executed until stability is obtained (using the idempotence condition). The $\delta _{B_1}$ denotes the standard dilation with the smallest unit structuring element $B_1$, $m\left( \bullet \right)$ is the mask image and *i* denotes the number of iterations.

Voxels of the resulting image, which are indicated by the mask shown in Fig. 4c, constitute a subset *D* of pixels helpful to determine the threshold value defined as:

4 $$\begin{aligned} th_k = \overline{D} + \sigma _D, \end{aligned}$$

where $\overline{D}$ denotes the mean value of *D*. The binary image (satisfying the condition $I(x,y,z)>th_k$) is subjected to the opening operation followed by morphological reconstruction to remove objects touching the rectangle borders in Fig. 4a. All operations are implemented in 3D.

The object removal procedure is performed until the number of remaining binary objects in each bounding box is larger than 1. When the volume of each object is comparable, both of them are considered. In other cases the smaller one is removed. This refers to the nephrectomy.

These binary objects serve as kidney seeds. Typically, their volume is slightly smaller than the kidney volume. The seeds are subjected to the markers generation step.

#### Generation of markers

**Fig. 5:**
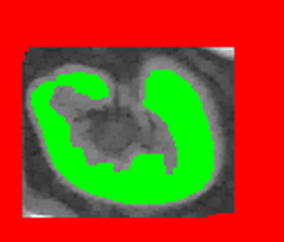
2D object (green) and background (red) markers required for marker-controlled watershed transform

Two markers are expected in the marker-controlled watershed transform. The first one, referred to as a object marker, is the image region obtained in the previous step. Figure 5 shows a single kidney, where green area reflects to object marker.

To obtain the background marker, for each slice including the kidney, a rectangular convex hull is applied. Moreover, to increase the kidney region a morphological dilation with a medium size structuring element is performed. The size of the structuring element depends on the kidney size and is set to 10% of the smallest size measured in the (*x*, *y*, *z*)-directions. Finally, the obtained image is inverted to mask the kidney background.

Both 2D markers are shown in Fig. 5. Due to 3D dilation the background marker in 2D may not be a rectangular. This improves its alignment to the kidney shape.

**Fig. 6:**
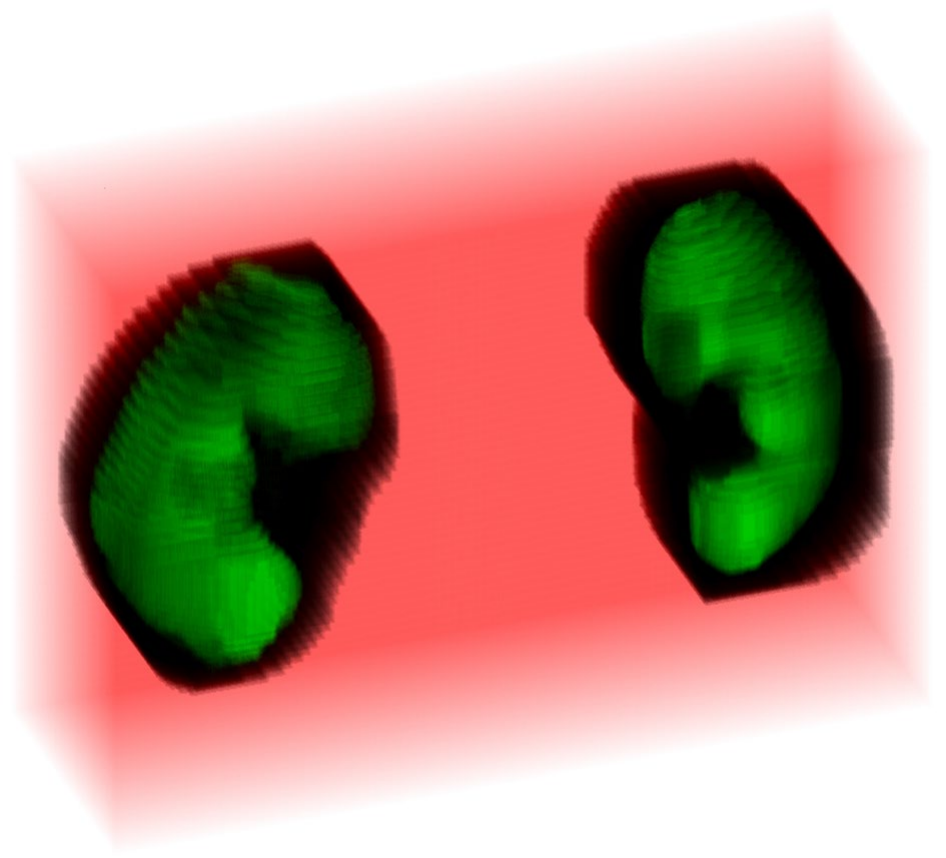
3D masks of the kidney (green) and background (red)

The spatial visualization of both markers is shown in Fig. 6. Red voxels refer to the background marker, green voxels correspond to the object marker. Dark voxels will be processed in the next stage to accurately extract the kidney edges.

#### Final kidney segmentation

The marker-controlled watershed transform (MCWT) [17–19] is applied in the last stage in kidney segmentation. MCWT is a modified version of the standard watershed transform enhanced by a reduction of the oversegmentation effect. The idea of this algorithm can be easily demonstrated based on a 2D gray level image or even 1D image profile (the latter will be used).

**Fig. 7:**
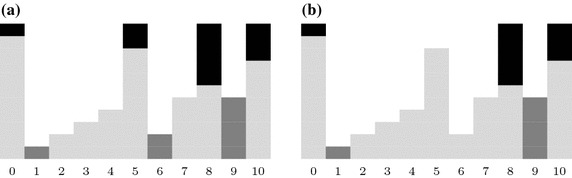
Comparison of **a** watershed transform and **b** marker-controlled watershed transform based on an image profile (x-axis pixel number, y-axis pixel intensity)

The watershed transform considers the image as a topographic relief, which is flooded by water. The flooding starts from all local minima in the image (dark gray boxes in Fig. 7a at positions 1, 6, 9). The water level successively raises, filling up all basins. At points where the water, coming from different basins, would meet, dams are built (black boxes in Fig. 7a at 0, 5, 8, 10). When the water level reaches the highest peak in the landscape, the process stops. The final dams arrangement represents image division into regions (the classic definition of image segmentation). Since watershed lines (dams) pass through the brightest pixels, the gradient magnitude image should be subjected to a further analysis.

The full analysis of all gradient local minima for the current application has two major drawbacks: the number of watershed basins is very high and watershed regions are located inside and outside of the kidney. To overcome these drawbacks the marker-controlled watershed algorithm was adopted.

In MCWT only some local minima are considered. Therefore, in Fig. 7b one local minimum (number 6) was skipped and the number of basins decreases. The dams are only assigned to pixels 0, 8 and 10. To indicate a desired local minima and to skip the unimportant ones, the object and background markers are employed.

The background marker blinded the irrelevant areas (red part in Fig. 6), while the object marker identified areas that should not be split (green part in Fig. 6). Therefore, only a small area is formed where the kidneys’ edges are searched (dark voxels in Fig. 6).

Due to the overlap of the kidneys’ edges and the dams, the gradient magnitude image is prepared. Since the gradient computation methods are noise sensitive, the average spatial filtering and morphological opening were performed. Both were implemented in 3D. The gradient magnitude is also calculated in 3D space according to equation:

5 $$\begin{aligned} \Vert \nabla I \Vert = \sqrt{\left( \frac{\partial }{\partial x} I \right) ^2 + \left( \frac{\partial }{\partial y} I \right) ^2 + \left( \frac{\partial }{\partial z} I \right) ^2}. \end{aligned}$$

**Fig. 8:**
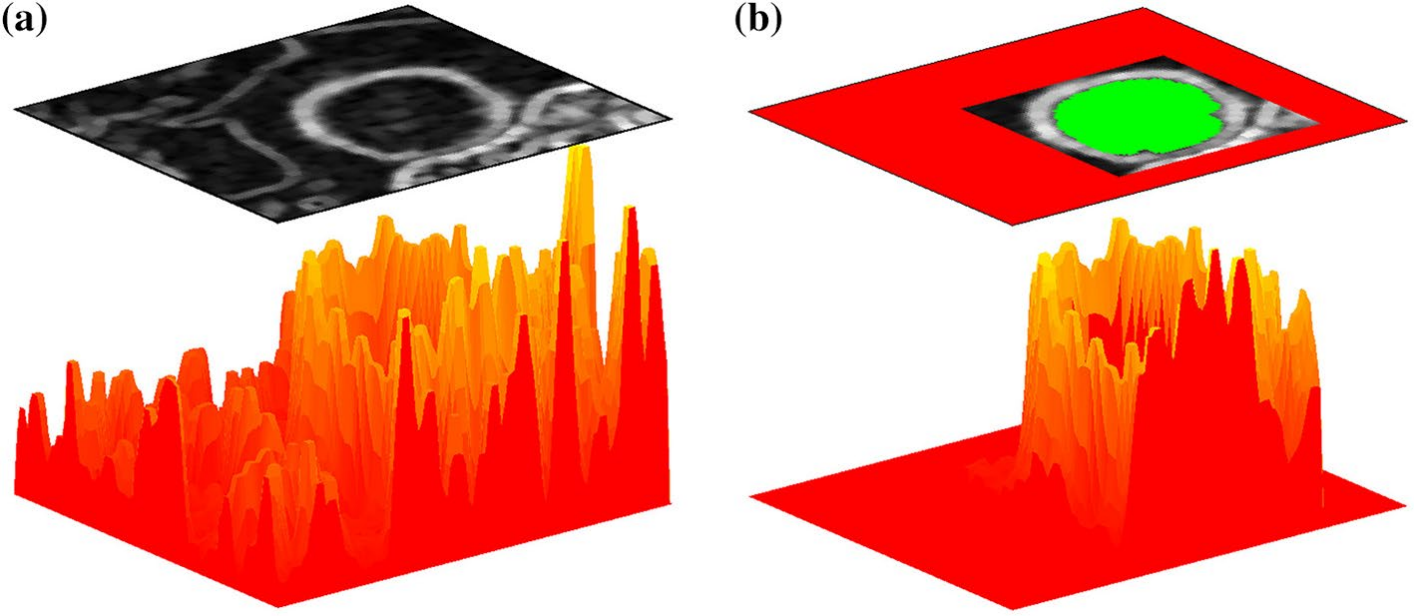
Image gradient magnitude: **a** full view, **b** view limited by object and background markers

An example of the gradient magnitude for a single slice and only one kidney is shown in Fig. 8a. In the upper part a 2D view is shown, while the bottom graph shows the topographic relief.

Despite a relatively small gradient window size, the gradient magnitude in Fig. 8a indicates the kidney edge as well as other structures. It is particularly visible when strong edge objects appear in the neighborhood. The use of markers allows only desirable edges to be obtained. This is shown in Fig. 8b. The area indicated by markers discovers only a corridor including the kidney edge. Thus, the watershed dams overlapped the kidney boundaries with high accuracy.

### Post-processing step

Since the watershed transform generated labeled images, the last step is a image binarization. To find labels corresponding to the kidney, the object marker image is reused. This image always indicates a region belonging to kidney but its volume is smaller than the desired kidney volume. However, the surface area of the region obtained from the watershed transform is more reliable.

**Fig. 9:**
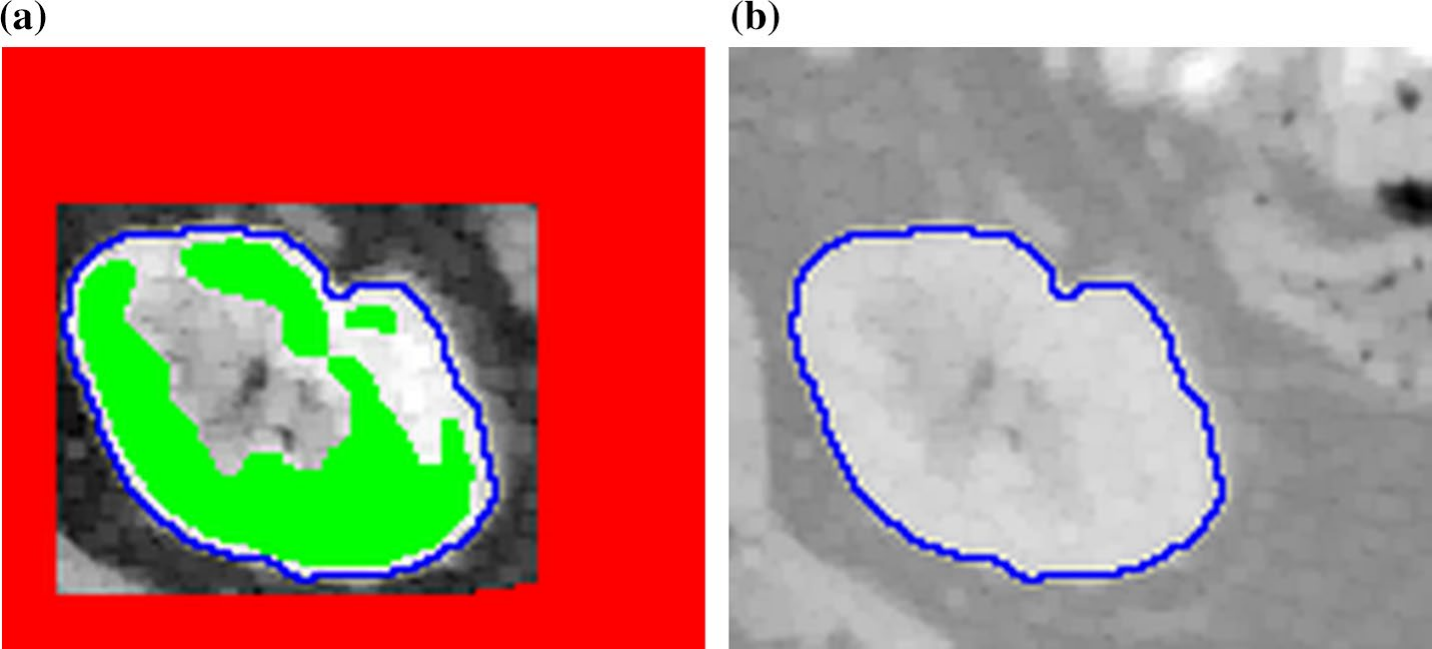
Comparison of **a** markers (red, green) and **b** final kidney delineation (blue)

Finally, morphological filtering (consecutive opening and closing) and hole filling is provided in order to smooth the kidney edges. The comparison of both markers with the computerized kidney delineation is shown in Fig. 9. It is clearly visible that the kidney edge is located in the space between markers. Although the object marker indicates only three disjointed kidney parts, the obtained delineation covers the real object edge.

### Statistical analysis

Statistical analysis was performed using the MATLAB environment, Version 2017a. The kidney segmentation quality has been assessed by the sensitivity:

6 $$\begin{aligned} Sens = \frac{TP}{TP + FN}, \end{aligned}$$

and the specificity:

7 $$\begin{aligned} Spec = \frac{TN}{TN + FP} \end{aligned}$$

coefficients. *TP*, *TN*, *FP*, *FN* denote the number of True Positive, True Negative, False Positive and False Negative voxel detections, respectively. The meaning of symbol notation is well known and it is as follows: TP refers to correctly identified as a kidney voxels, FP incorrectly identified voxels, TN correctly rejected and FN incorrectly rejected voxels. In the following section of the paper, both measures (sensitivity and specificity) are presented in a percentage scale.

Moreover, the segmentation results are validated by the Dice index:

8 $$\begin{aligned} D = \frac{2 \cdot TP}{2 \cdot TP + FP + FN}, \end{aligned}$$

and the Jaccard index:

9 $$\begin{aligned} J = \frac{D}{2 - D}. \end{aligned}$$

Both the Dice index and Jaccard index are considered in the percentage scale.

Finally, the dispersion between manual or semi-automatic delineations and segmentation results is evaluated by Cohen’s Kappa [20] measure defined as:

10 $$\begin{aligned} \kappa = \frac{Acc - randAcc}{1-randAcc} \end{aligned}$$

where accuracy (*Acc*) is an observational probability of agreement and random accuracy (*randAcc*) is a hypothetical expected probability of agreement under an appropriate set of baseline constraints [21]. Accuracy can be written as:

11 $$\begin{aligned} Acc = \frac{TP+TN}{TP+TN+FP+FN}, \end{aligned}$$

while random accuracy as:

12 $$\begin{aligned} randAcc = \frac{(TN+FP)\cdot (TN+FN)+(FN+TP)\cdot (FP+TP)}{(TP+TN+FP+FN)^2}. \end{aligned}$$

The $\kappa$ value can be interpreted as shown in Table 2 [22].

**Table 2:** Interpretation of $\kappa$ value

$\kappa$, %	Strength of agreement
100–81	Very good
80–61	Good
60–41	Moderate
40–21	Fair
20–0	Poor

## Results

### Reference data set

Due to the large database employed for evaluation, a manual delineation of all slices is very time consuming. Since no commercial tool dedicated to accurate and fast kidney segmentation is available, a semi-automated 3D Slicer [23, 24] procedure has been adopted. The Editor Module of 3D Slicer includes the Level Tracing Effect tool. It delineates the boundary of segmented structures in 2D and snaps it on a user request. The segmentation with Level Tracing Effect tool has been carried out under the permanent visual control of the expert. Since the expert’s impact on the contour extracted is smaller, the delineation has been evaluated by comparing the results with a manual segmentation performed by a medical expert on a limited set of CT studies.

**Fig. 10:**
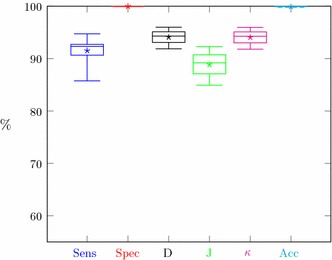
Comparison between manual and semi-automatic delineations

The comparison of the delineations performed manually and semi-automatically for 12 is shown in Fig. 10. In addition to the typical values associated with box plots (such as minimum— lower whisker, maximum—upper whisker, the first and third quartiles values—the box span, median—vertical line within the box) the mean value has been marked (isolated points in the figures).

**Fig. 11:**
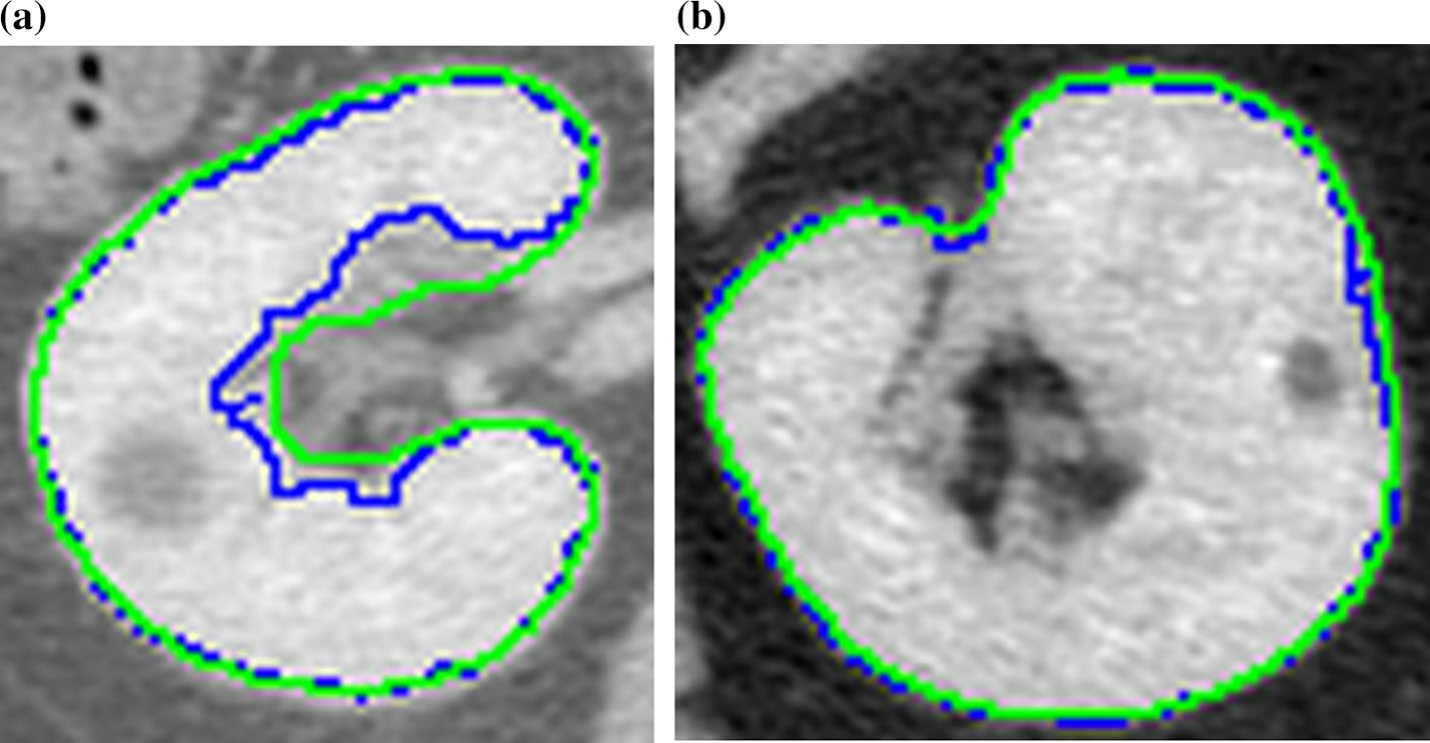
Examples of segmentation delineated manually by an expert (green) and by the Level Trace Effect tool (blue) for **a** left, and **b** right kidney

A discrepancy between the manual and semi-automatic delineation can be noticed in the vascular cavity region (Fig. 11a), yet the external edge delineations are well aligned (Fig. 11b).

Findings demonstrate a very high convergence of manual and semi-automatic delineation technique. Mean values of sensitivity, specificity, Dice, Jaccard, Cohen’s $\kappa$ and accuracy are 91.49, 99.98, 94.11, 88.90, 94.07 and 99.92%, respectively. These metrics are obtain based on 12 CTs with both manual and semi-automatic delineations. The lack of higher consistency between both delineation techniques is mainly due to differences in vascular cavity segmentation (Fig. 11). Since both delineations are highly comparable, the Level Tracing Effect tool can be successfully used to generate the gold standard in further evaluation stages.

**Fig. 12:**
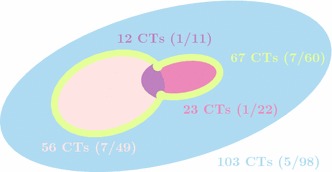
Reference database with number of CTs (physiological/pathological cases) where cases delineated manually has been highlighted using  color, semi-automatic , both manually and semi-automatic  and cases undelineated using  color

The detailed summary of reference database is presented in Fig. 12. For further evaluation three reference data sets with manual and semi-automatic segmentation as well as undelineated kidneys are employed. The validation was performed in two steps. First, the segmentation quality was assessed by comparing the automated segmentation results to the delineated kidney edges. Secondly, the Altman classes were used in order to assign each segmentation result to one of the edges.

### Quality of kidney segmentation

**Fig. 13:**
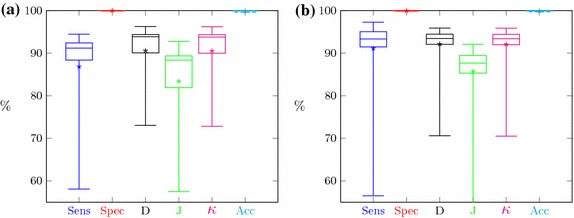
Segmentation quality coefficient for CTs with: **a** manual delineations, **b** semi-automatic delineations

The evaluation of the kidney segmentation quality is performed in two steps. The first step compares the segmentation results with the manual, expert delineations. The evaluation based on 23 cases is shown in Fig. 13a. The segmentation quality expressed by the aforementioned measures for the majority of cases is relatively high (averages reach 90%). Single cases feature small index values. This is caused by a small over- or under-segmentation effect or the aforementioned differences in the vascular cavity segmentation. Moreover, the segmentation results of 5% of cases becomes outliers.

The second step of the evaluation procedure is based on a comparison of the segmentation results with the semi-automatic expert delineations obtained with the Level Tracing Effect tool. The accuracy indices of 56 CT studies are shown in Fig. 13b.

**Fig. 14:**
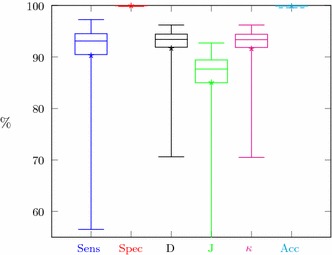
Segmentation quality coefficient for CTs with any delineations

Segmentation results for all CT series with manual or semi-automatic delineation (67 cases) have been summarized in Fig. 14.

### Group quantitative assessment

Based on Cohen’s $\kappa$ and the Altman rules [22], all delineated cases have been classified to one of five groups given in Table 3.

**Table 3:**
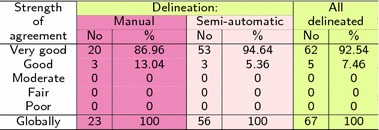
Classification of the segmentation results for delineated cases

In order to evaluate the remaining CT exams with neither manual nor semi-automatic delineation, another test was performed. It runs in two steps. The first step is the training procedure. Delineated cases were used to teach medical experts the Altman rules [22]. Medical cases from the training database consisting of 67 CTs were assigned to one of five groups. The assignment was based on $\kappa$ coefficient value. The obtained results are shown in Table 3 in three categories: delineated manually, delineated semi-automatic and delineated manually or semi-automatic. Letters A, B and C are refer to subsequent medical experts.

After the training procedure, all CTs have been classified by the medical expert into five groups according to Altman rules (Table 4). Since 103 CTs have no delineations, a medical expert validation procedure has been performed visually.

**Table 4:**
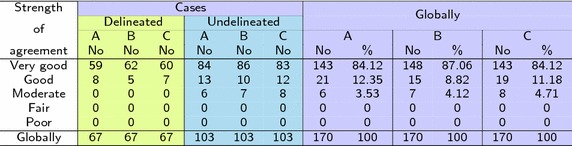
Classification of the segmentation results for delineated cases

### Graphical results

**Fig. 15:**
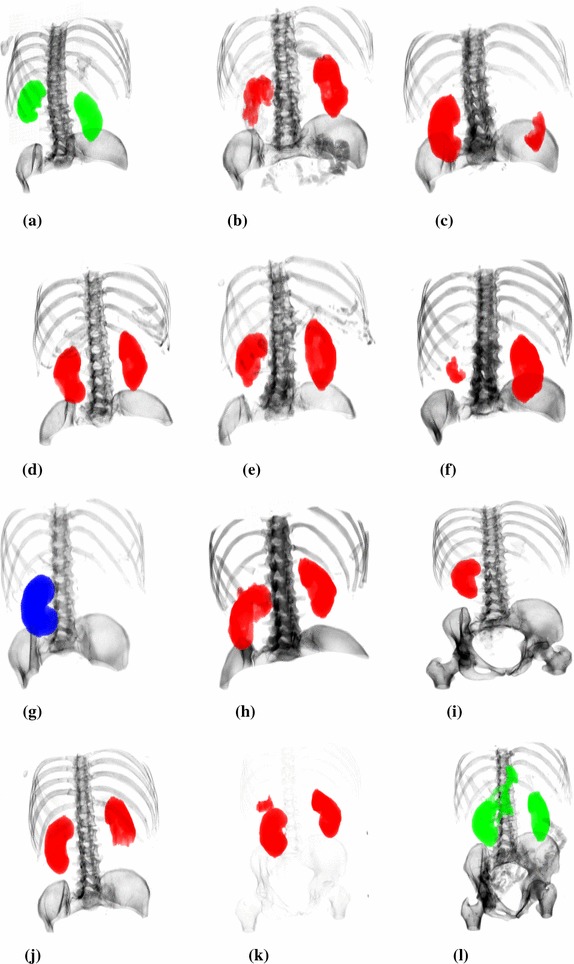
3D view of the segmentation results (physiological cases are displayed in green, pathological cases are displayed in red, while nephrectomy is displayed in blue). **a** Case 751_11, **b** case 1167_10, **c** case 1480_10, **d** case 95_13, **e** case 1649_13, **f** case 1675_13, **g** case 1070_12, **h** case 2111_13, **i** case 3322_11, **j** case 1223_11, **k** case 2802_13, **l** case 1972_12

The graphical representation of the segmentation results in 3D view is shown in Fig. 15. Subsequent columns represent cases of different Altman classes. The following pathologies are included: right kidney tumor (Fig. 15b), left kidney tumor and both kidney cysts (Fig. 15c), tumor and cysts in right kidney (Fig. 15d), tumors in both adrenal glands and the right kidney after surgery (Fig. 15e), left kidney adenoma and cysts and right kidney cirrhosis (Fig. 15f), right kidney tumor and hematoma (Fig. 15h), both kidney focal lesions (Fig. 15i), left kidney tumor (Fig. 15j) and left kidney focal lesions and nephrolithiasis (Fig. 15k). Moreover, Fig. 15g presents a case after nephrectomy (left kidney was removed).

**Fig. 16:**
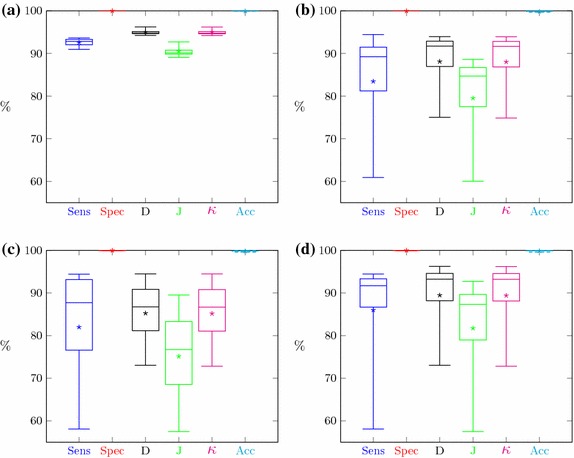
The segmentation quality measures for cases from Fig. 15: **a** left column, **b** middle column, **c** right column and **d** for all cases from Fig. 15

The CT series visible in Fig. 15 have been selected only from cases delineated manually. Thus, determination of all quality measures for these cases is possible. Results for three Altman classes are presented in Fig. 16.

### State-of-the-art comparison

The study described in this paper exceeds the scope of work presented in [1, 2, 5–8, 10–12]. The presented method was developed, tested and validated on a large set of CT examinations (170 CTs) containing clinically normal and abnormal kidneys. In order to assess the performance of the presented method versus the state-of-the-art, one would need either algorithm sources or the image data with manual delineations from the respective studies. Since none of these conditions are fulfilled, the assessment was performed based on the accuracy, Dice indices, sensitivity and specificity reported by authors. Furthermore, in order to compare with research presented in [5] False Positive Volume Fraction index defined as:

13 $$\begin{aligned} FPVF = \frac{FP}{TN+FP} \end{aligned}$$

was used. Analogously, for Zollner et al. [11] the similarity measure:

14 $$\begin{aligned} Sim = 1 - \frac{|FN-FP|}{2 \cdot TP + FN + FP} \end{aligned}$$

is introduced. A detailed quality comparison is shown in Table 5. The obtained results for the presented algorithm are better than most of the reported in the literature.

**Table 5:**
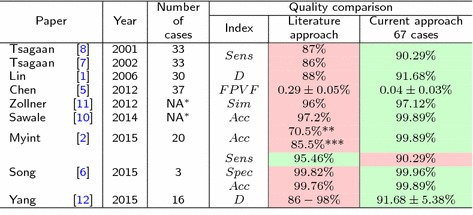
State-of-the-art comparison

$$^*$$ Not available, $$^{**}$$ for region growing, $$^{***}$$ for gradient-based

## Discussion

Automatic or semi-automatic kidney segmentation has been investigated by different research groups in the field. The underlying building blocks of these algorithms consist of region of interest extraction, region growing [1], edge detection [2] or more complex algorithms such as graph cut, GrowCut [5], Fuzzy C-Means, level-set [3, 4] and many others [6, 13–15, 25–27]. Most algorithms in the literature usually incorporate over a dozen (no more than 37) CTs portal venous phase into their validation. In the current study, a fully automatic kidney segmentation approach is adapted to clinical conditions. A large database and variety of medical acquisition protocols have been subjected to the analysis. The applied algorithms in both processing steps provide satisfactory results. The first stage of rough kidney segmentation uses mathematical morphology operations and algorithms, in particular morphological image reconstruction. In this way, object and background markers are obtained. These images are crucial for the marker controlled watershed transform. Consequently the rough segmentation results are matched to the real kidney edges. The current study database includes 170 cases whereas 67 are delineated by a medical expert manually (23 CTs) or semi-automatic (56 CTs). Since the semi-automatic delineation is less accurate yet faster and less time-consuming than the manual outline, its robustness has to be evaluated. Thus, the accuracy of the semi-automatic delineation tool (Level Tracing Effect from Slicer3D) was identified.

Segmentation quality assessment was provided separately for manual and semi-automatic delineation and globally for all cases delineated in any way. The results for both separable groups (Fig. 13) showed their high similarity. The majority of medical cases have high segmentation metrics. Boxes in Fig. 13 are small and located at about 90%. Specificity and accuracy exceed 99%, sensitivity, Dice index, and Cohen’s $\kappa$ exceed 90% and the lowest value above 85% is a Jaccard coefficient. Only isolated cases deviate from mentioned values as evidenced by lower whiskers. Since the semi-automatic group is larger, the range of metric values is slightly smaller than for the group with manual delineations (boxes in Fig. 13b are smaller than in Fig. 13a). Due to the high similarity of the results for both delineation groups, the global results (Fig. 14) also show similar properties. These findings demonstrate the high efficiency of this kidney segmentation approach both for physiological and pathological cases, so the method appears quite robust in clinical applications. These results also prove that the use of semi-automatic expert delineations are reasonable.

The Cohen’s $\kappa$ index can be interpreted as shown in Table 2. This gives a basis to classify delineated cases between five groups with different qualities of segmentation. The classification has been performed separately for manual and semi-automatic delineations, and then together for all available delineations (Table 3). All delineated cases belong to the first two quality groups called ‘Very good’ and ‘Good’, wherein a ‘Very good’ group size exceed 92%.

Next, the assessment procedure was conducted since not all medical cases were delineated by an expert. After a training procedure (conducted based on delineated cases) three different experts classified all available medical cases between Altman classes. Their assessments were very similar to each other. Each expert’s findings oscillate around values resulting from the $\kappa$ index. Moreover, one expert (expert B) classified delineated cases identically as it resulted from coefficient $\kappa$ (compare green columns in Tables 3, 4). It can be stated that such a form of assessment is justified in the absence of delineations. These findings confirm both the reliability of the study and the high effectiveness of the method. Almost 85% of 170 cases were classified as ‘Very good’. Whereas the remaining 15% included ‘Good’ and ‘Moderate’ classes, wherein the majority were ‘Good’. Only a few percent of cases were included in category ‘Moderate’.

The proposed numerical indicators and the graphical results confirm the high efficiency of the method. This concerns both planar (Fig. 11) and spatial images (Fig. 15). Decreases in the coefficients of quality measure often resulted from differences in the kidney vascular cavities interpretation (Fig. 11). There were also cases with leaks to neighboring vessels (Fig. 15l) or organs (Fig 15h). Also, the opposite cases with incomplete segmentation occurred (Fig 15b, c, f, i, j). The reason is often a surgical intervention or some pathologies, i.e. cirrhosis, tumors. The worst segmentation cases has been presented in Fig. 15, although they represent a small percentage of the entire dataset. This figure does not reflect the true proportion between worse- and better-segmented cases.

These findings also seems competitive with respect to the state-of-the-art (Table 5). In most cases the quality indices are better than presented in the literature. Only the specificity reported in [6] is higher than in the presented solution. However, the reported value is based on the evaluation of 3 cases, thus may not be representative. Moreover, state-of-the-art approaches were not verified with such a large and comprehensive clinical database. Taking control of such a large number of differentiated medical cases has been a big challenge.

## Conclusions

The current research develops a fully automatic kidney segmentation approach as a 3D extension of marker-controlled watershed transform. The expected marker images (object and background) are generated automatically based on image geometry and brightness. The kidney walls are located by the 3D watershed transform.

Findings based on large database demonstrate high values of segmentation quality metrics (accuracy over 99% and mean Dice and Cohen’s $\kappa$ over 91%). Analogously, an expert assessment indicates its usefulness under clinical conditions. These relatively high metrics were obtained despite the database size and variety of CT studies performed by different medical staff and various CT scanners. Neither patient conditions nor cases have been selected. This makes the testing environment rather difficult. Nevertheless, the outcome was classified by the experts as ‘Very good’, ‘Good’ or ‘Moderate’ and is being employed in generating a patient related model for the image-guided minimally invasive abdominal surgery.

## Abbreviations

2D: two-dimensional
3D: three-dimensional
CT: computed tomography
MRI: magnetic resonance imaging
OCT: optical coherence tomography
HU: hounsfield units
HMAX: H maxima transform
WT: watershed transform
MCWT: marker-controlled watershed transform
TP: True Positive
TN: True Negative
FP: False Positive
FN: False Negative
